# A Pigeon-Derived Sub-Genotype XXI.1.2 Newcastle Disease Virus from Bangladesh Induces High Mortality in Chickens

**DOI:** 10.3390/v13081520

**Published:** 2021-08-01

**Authors:** Mohammed Nooruzzaman, Lalita Rani Barman, Tanjin Tamanna Mumu, Emdadul Haque Chowdhury, Kiril M. Dimitrov, Mohammad Rafiqul Islam

**Affiliations:** 1 Department of Pathology, Faculty of Veterinary Science, Bangladesh Agricultural University, Mymensingh 2202, Bangladesh; mohammed.nooruzzaman@bau.edu.bd (M.N.); lalitalalita2002@hotmail.com (L.R.B.); tanjinmumu@bau.edu.bd (T.T.M.); emdad@bau.edu.bd (E.H.C.); 2 Texas A&M Veterinary Medical Diagnostic Laboratory, 483 Agronomy Rd, College Station, TX 77843, USA

**Keywords:** Newcastle disease virus, genotype XXI.1.2, pigeons, pathology, Bangladesh, PPMV-1, pathogenicity

## Abstract

Newcastle disease virus (NDV) is a significant pathogen of poultry; however, variants also affect other species, including pigeons. While NDV is endemic in Bangladesh, and poultry isolates have been recently characterized, information about viruses infecting pigeons is limited. Worldwide, pigeon-derived isolates are commonly of low to moderate virulence for chickens. Here, we studied a pigeon-derived NDV isolated in Bangladesh in 2010. To molecularly characterize the isolate, we sequenced its complete fusion gene and performed a comprehensive phylogenetic analysis. We further studied the biological properties of the virus by estimating mean death time (MDT) and by experimentally infecting 5-week-old naïve *Sonali* chickens. The studied virus clustered in sub-genotype XXI.1.2 with NDV from pigeons from Pakistan isolated during 2014–2018. Deduced amino acid sequence analysis showed a polybasic fusion protein cleavage site motif, typical for virulent NDV. The performed in vivo pathogenicity testing showed a MDT of 40.8 h, and along with previously established intracerebral pathogenicity index of 1.51, these indicated a velogenic pathotype for chickens, which is not typical for pigeon-derived viruses. The experimental infection of chickens resulted in marked neurological signs and high mortality starting at 7 days post infection (dpi). Mild congestion in the thymus and necrosis in the spleen were observed at an advanced stage of infection. Microscopically, lymphoid depletion in the thymus, spleen, and bursa of Fabricius were found at 5 dpi, which progressed to severe in the following days. Mild to moderate proliferation of glial cells was noticed in the brain starting at 2 dpi, which gradually progressed with time, leading to focal nodular aggregation. This study reports the velogenic nature for domestic chickens of a pigeon-derived NDV isolate of sub-genotype XXI.1.2. Our findings show that not all pigeon-derived viruses are of low virulence for chickens and highlight the importance of biologically evaluating the pathogenicity of NDV isolated from pigeons.

## 1. Introduction

Newcastle disease (ND) is caused by virulent strains of *avian orthoavulavirus 1* (AOAV-1), also known as avian paramyxovirus 1 (APMV-1) or Newcastle disease virus (NDV, used hereafter), belonging to genus *Orthoavulavirus* of the family *Paramyxoviridae* [1]. NDV is an enveloped virus containing a negative-sense, single-stranded RNA genome [2] of approximately 15.2 kb that encodes for six major proteins: nucleocapsid (N) protein, phosphoprotein (P), matrix (M) protein, fusion (F) protein, haemagglutinin-neuraminidase (HN), and large RNA-dependent RNA-polymerase (L). All NDV isolates belong to a single serotype and are divided into three main pathotypes based on their pathogenicity for chickens (listed in order of decreasing virulence): velogenic, mesogenic, and lentogenic (includes asymptomatic enteric) [3,4]. Velogenic viruses produce high mortality, particularly in immunologically naïve chickens, and are further divided into viscerotropic velogenic NDV (vvNDV), causing haemorrhagic lesions in the gastrointestinal tract, and neurotropic velogenic NDV (nvNDV), which produce predominantly neurological but also respiratory signs. Mesogenic viruses are of moderate virulence, causing mostly respiratory disease, whereas lentogenic viruses are of low virulence or avirulent and cause no clinical disease to mild enteric, respiratory, or subclinical infection mostly in younger birds [5]. The two surface glycoproteins F and HN play a key role in the virulence of NDV [6,7]. The F protein and specifically its cleavage site amino acid sequence is known to be the major determinant of NDV virulence [8,9]. Strains of NDV (vNDV) that possess a cleavage site with multiple basic amino acids at the C-terminus of the F2 protein with motif ^113^R-Q-K/R-R^116^ and phenylalanine at the N-terminus of the F1 protein (residue 117) are considered virulent [4,10].

Based on their full-length F-gene coding sequences, NDV isolates are classified into multiple genotypes and sub-genotypes under two main groups, namely class I and class II [11,12]. Class I viruses belong to a single genotype, are mostly avirulent, and have predominantly been detected in waterfowl and shorebirds [13,14]. Viruses of class II NDV are currently classified into 21 (I-XXI) genotypes, some with multiple sub-genotypes [11]. Class II viruses have been detected in both domestic poultry and wild birds worldwide and have caused at least five ND panzootics [15].

NDV of class II genotypes VI and XXI (the latter being newly formed and separated from VI) have been associated with most ND outbreaks in pigeons and doves [16]. However, these species have also been affected by viruses of genotype VII [11], which commonly infect poultry [17]. Pigeon paramyxoviruses 1 (PPMV-1) are antigenic variants of NDV adapted to birds of the family *Columbidae*. These viruses have been previously distinguished from other NDV by a panel of monoclonal antibodies (mAb) [18] and have been reported to form sub-genotype VI.1.1 (formerly designated as VIb) [12,19]. This mAb panel is neither widely available nor commonly used, and not all viruses from genotype VI (and genotype XXI that was created from viruses formerly belonging to genotype VI) have been shown to be PPMV-1. Of note, while the term PPMV-1 has initially been used to designate only the viruses of sub-genotype VI.1.1, it has also been often used for all viruses from genotype VI without evidence that they are indeed PPMV-1 [20,21].

With the rapid development and availability of molecular methods, these are now almost universally utilized for characterization of avian paramyxovirus isolates. Although many pigeon-derived viruses have been found to possess multiple basic amino acid residues at the F-protein-cleavage site, which is typical for virulent NDV, experimental inoculation of many of these viruses revealed variable clinical outcomes in chickens [22,23,24,25,26]. While PPMV-1 commonly produce high morbidity and mortality in pigeons, infections in chickens often present with mild to moderate neurological signs [23,24,27] or even absence of clinical disease [22,25,28]. These findings are also supported by establishing the intracerebral pathogenicity index (ICPI), with many pigeon-derived viruses having values below 1.5 [28,29,30,31]. It has previously been reported that continuous circulation of pigeon viruses in poultry could lead to generation of strains virulent to poultry [32,33,34] and, although only occasionally, their potential to cause disease in chickens has been demonstrated [23,27,35,36].

Velogenic viscerotropic NDV is endemic in Bangladesh, causing continuous outbreaks in commercial as well as backyard poultry [37,38]. Our recent studies identified and characterized velogenic NDV isolates from several outbreaks in chickens and pigeons in Bangladesh [39,40,41,42]. However, detailed molecular and phylogenetic characterization and pathogenicity studies in chickens of pigeon-derived Bangladeshi NDV isolates are not available. Here, we aimed to molecularly investigate a Bangladeshi pigeon-derived NDV by sequencing its complete fusion protein gene and performing a comprehensive phylogenetic analysis to establish its relationship to other viruses based on the unified NDV classification system. We further aimed to study the biological properties of the virus by estimating mean death time (MDT) and by experimentally infecting 5-week-old naïve chickens.

## 2. Materials and Methods

### 2.1. Ethics Statement

All applicable national and institutional guidelines for the care and use of animals were followed. The study was carried out in accordance with the recommendation of the Ethical Standard of Research Committee of Bangladesh Agricultural University, Mymensingh. The utilized protocol and procedures were reviewed and approved by the Ethical Standard of Research Committee (Ref. No. BAURES/ESRC/693/2020; Dated: 10.06.2020).

### 2.2. Virus

A pigeon-derived NDV isolate pigeon/Bangladesh/BD-P01/2010 (referred to as BD-P01 hereafter) was retrieved from the repository of the Department of Pathology, Bangladesh Agricultural University. The virus was originally isolated from an outbreak in a small backyard flock (12 birds) of 6-month-old domestic pigeons in 2010 [39]. The birds were reared in wooden boxes in a rural household, and chickens were also raised in the same backyard. The single affected pigeon was found highly lethargic and anorexic before death, and gross examination showed haemorrhages in the proventriculus. For inoculum preparation, the virus was propagated in 9-day-old specific pathogen free (SPF) embryonating chicken eggs (ECE) via allantoic cavity route, and the infected allantoic fluid was harvested following death of the embryo. The identity of the virus in the infected allantoic fluid was confirmed by RT-PCR, as described previously [40]. The inoculum was also tested and found negative for avian influenza (AI), infectious bronchitis virus (IBV), and infectious bursal disease virus (IBDV) using virus-specific RT-PCR, as described elsewhere [43,44,45].

### 2.3. Pathogenicity Testing

The pathogenicity of the pigeon isolate was assessed by estimating the mean death time (MDT) of embryonated chicken eggs following a standard procedure [5]. The ICPI of the isolate was previously established (ICPI = 1.51) [39].

### 2.4. Sequencing

Viral RNA was extracted by using the MagMAX™-96 viral RNA isolation kit and a KingFisher™ Magnetic Particle Processor (ThermoFisher Scientific, USA). For F-gene amplification, SuperScript^®^ III One-Step RT-PCR System with Platinum^®^ Taq DNA polymerase (Life Technologies, USA) was used following the manufacturer’s recommendations. The F-gene coding sequence was amplified using two overlapping RT-PCR with primer pairs NDVF13-F1 5′-GAC GCA ACA TGG GCT CCA RAY CTT-3′, NDVF13-R1 5′-GGC AAA CCC TCT GGT CGT GCT YAC-3′, and NDVF13-F2 5′-TTG GGA AAA TGC AAC AGT TTG-3′, NDVF13-R2 5′-GCAT TCA CCT TTC ATC TGC GTT CA-3′ [46]. The RT-PCR product was subjected to electrophoresis in 1% agarose gel. The DNA band was excised, purified, and sequenced by a commercial laboratory (1st Base, Malaysia). The raw sequence data were assembled and edited using BioEdit (www.mbio.ncsu.edu/BioEdit/bioedit.html).

### 2.5. Collection of Sequences

The dataset of complete F-gene sequences provided by the international consortium that published the current NDV classification system [11] was used in this study (dataset deposited in GitHub at https://github.com/NDVconsortium/NDV_Sequence_Datasets). As of 30 October 2020 the dataset contained 1901 sequences. All collected sequences were aligned using Multiple Alignment with Fast Fourier Transformation (MAFFT v7.4.50) [47] as implemented in Geneious Prime v.2021.0.1 (Biomatters Ltd., New Zealand). Additional sequences (*n* = 50), submitted to GenBank after the dataset was created, were downloaded and added to the alignment (as of 30 March 2021).

### 2.6. Evolutionary and Phylogenetic Analyses

To study the evolutionary relationship between the BD-P01 and viruses isolated in other geographical regions, phylogenetic analyses were performed. The estimates of average nucleotide distances were inferred using MEGA6 [48]. Analyses were conducted using the Maximum Composite Likelihood model [49]. The rate variation among sites was modelled with a gamma distribution (shape parameter = 1).

Next, utilizing MEGA6, the corrected Akaike information criterion was estimated using all possible models. The general time-reversible (GTR) model [50] was identified as best goodness of fit, and a maximum-likelihood tree with 1000 bootstrap replicates was built using RaxML version 8.2.12 [51]. Evolutionary rate differences among sites were modelled with discrete Gamma distribution (Γ), and the rate variation model allowed for some sites to be evolutionarily invariable (I). The RaxML tree was constructed through the CIPRES Science Gateway [52]. The tree was inferred using the complete class II full-length F-gene alignment (*n* = 1952, including the sequence obtained here). The tree was visualized using FigTree v1.4.2 (http://tree.bio.ed.ac.uk/software/figtree). The taxa names include a Roman–Arabic numeral representing the respective genotype for each isolate, the GenBank accession number, host name (if available), country of isolation, isolate designation, and year of isolation. The criteria put forth by Dimitrov and co-authors [11] based on the phylogenetic topology and evolutionary distances between different taxonomic groups were used for sub/genotype identification.

Bayesian time scaled analysis was conducted by the Bayesian Markov Chain Monte Carlo (BMCMC) method implemented in BEAST v1.10.4 [53] program utilizing a subset of all full-fusion gene sequences (*n* = 383) of genotypes VI, XX, and XXI (representing all former genotype VI isolates, commonly considered to be pigeon related). General time-reversible model with gamma distribution nucleotide substitution were applied (GTR+ Γ4) [50,54]. Relaxed clock model (uncorrelated lognormal distribution) [55] with exponential growth demographic model was utilized. An input file for BEAST analysis was prepared using Bayesian evolutionary analysis utility (BEAUTI) tool v.1.10.4, and the sequences were annotated with year of collection. Three independent chains were run through the CIPRES Science Gateway using BEAGLE library [56] to get output of 100,000 trees from each run. Convergence was assessed in Tracer v1.7.1 program [57]. The trees from each run were combined using LogCombiner v.1.10.4 (burn in 30%), and Maximum Clade Credibility trees were generated using the Tree Annotator program v.1.10.4 from the BEAST package. The FigTree v1.4.2 tool was used for the visualization of the annotated tree.

### 2.7. Chickens

A total of 53 one-day-old *Sonali* chickens (crossbred of Fayoumi and Road Island Red chickens) were obtained from the Central Poultry Farm, Mirpur, Dhaka, Bangladesh. The chickens were reared in relative isolation without vaccination and provided feed and water with *ad libitum* access. At 28 days of age, chickens were bled, and the sera were separated. The serum samples were subjected to haemagglutination inhibition (HI) test following standard procedure [10]. None of the samples had detectable level of NDV-specific antibodies. At 32 days of age, the birds were divided into two groups (infected and control) and placed into separate houses for acclimatization.

### 2.8. Experimental Infection

At 35 days of age, the chickens from the experimental group (*n* = 31) were inoculated with 10^6^ EID_50_/0.1 mL of the BD-P01 virus. Each bird received 100 µL inoculum through the intraocular (50 µL) and intranasal (50 µL) routes. The control birds (*n* = 22) received 100 µL of uninfected allantoic fluid via the same routes. All birds were closely observed for clinical signs during the post-inoculation period, and morbidity and mortality were recorded daily. Birds that showed severe clinical signs, stopped eating or drinking, or remained recumbent were euthanized and reported as dead on the next day for the calculation of survival curve and average death time. Prism v.7.03 (GraphPad Software Inc., La Jolla, CA, USA) software was used to analyze a survival curve using the log-rank (Mantel–Cox) test.

### 2.9. Necropsy, Sample Collection and Histopathological Examination

All euthanized birds were necropsied, and gross lesions were recorded. At 1, 2, 3, 5, 7, and 9 days post infection (dpi), three birds per group (euthanized when sick or sacrificed) were necropsied, and gross lesions were recorded. Birds found dead at inspection were also examined. At necropsy, tissue samples from the Harderian gland, brain, thymus, spleen, liver, kidney, heart, trachea, lungs, proventriculus, intestine, caecal tonsils, and bursa of Fabricius were collected in 10% neutral buffered formalin. Next, fixed tissue samples were processed, sectioned, and stained with routine haematoxylin and eosin staining methods, as described previously [58]. The slides were examined under photomicroscope (ZEISS Primo Star, Germany).

## 3. Results

### 3.1. Pathogenicity Testing

The pathogenicity testing of the pigeon isolate was performed in embryonating chicken eggs. The BD-P01 virus showed a mean embryo death time (MDT) of 40.8 h. Such MDT (<60) is typical for viruses that are of high virulence for chickens [3]. This result is in agreement with the estimated ICPI of 1.51. While lentogenic viruses commonly have ICPI below 0.7 and MDT above 120 h, these indexes are MDT of 60–90 h and ICPI of 0.7–1.5 for mesogenic viruses and MDT below 60 h and ICPI above 1.5 for velogenic viruses [4].

### 3.2. Molecular Characterization and Classification

The complete F-gene coding sequence of the BD-P01 isolate was obtained and analyzed (submitted to GenBank and available under accession number JX028552). Analysis of the deduced amino acid sequence at the fusion protein cleavage site of BD-P01 revealed presence of multiple basic amino acid residues at the C-terminus of the F2 protein with motif ^113^R-Q-K/R-R^116^. The amino acid at the N-terminus of the F1 protein (residue 117) was deduced to be phenylalanine. Such cleavage site is specific for virulent viruses based on criteria utilized by OIE to assess virulence of NDV isolates [10].

**Figure 1:**
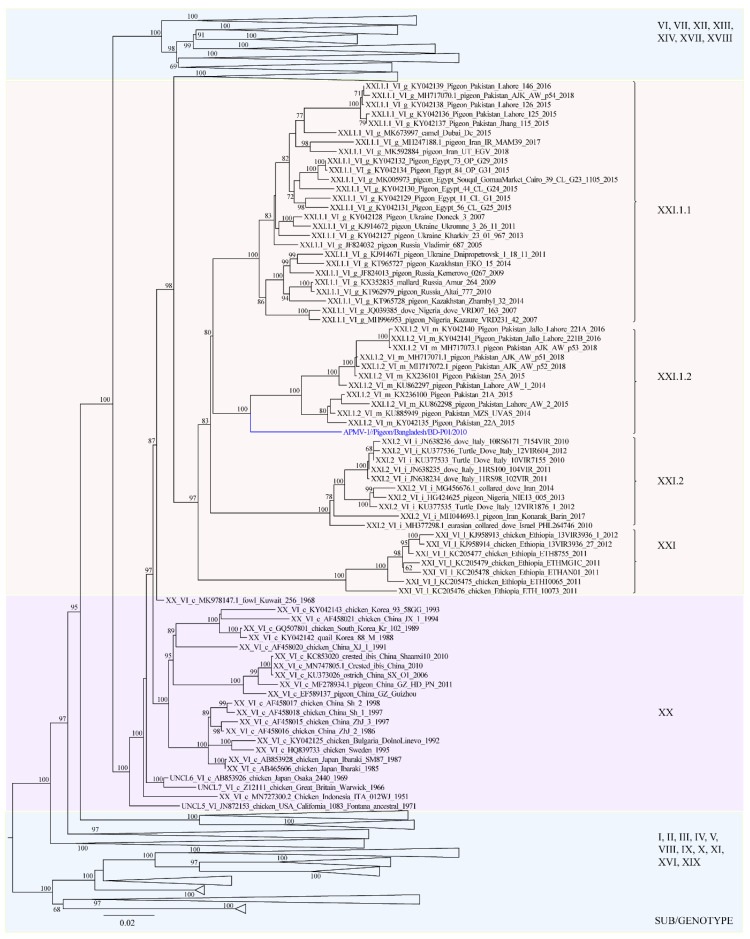
Phylogenetic analysis based on full-length nucleotide sequence of the fusion gene of isolates representing Newcastle disease virus class II (*n* = 1952). The evolutionary history was inferred by using RaxML [51] and utilizing the maximum-likelihood method based on the general time-reversible model with 1000 bootstrap replicates. A discrete gamma distribution was used to model evolutionary rate differences among sites, and the rate variation model allowed for some sites to be evolutionarily invariable. For imaging purposes, only isolates from genotype XX and XXI (*n* = 78) are shown; all other genotypes are collapsed (the full tree is available in Supplementary Figure S1). The tree is drawn to scale, with branch lengths measured in the number of substitutions per site. The Bangladeshi isolate BD-P01 is highlighted in blue color. There were a total of 1649 positions in the final dataset. Only bootstrap values above 60 are shown.

The nucleotide distances of the BD-P01 virus to related NDV from genotype XX, XXI, and genotype VI were estimated (Table 1). The analysis identified that the viruses most closely related to BD-P01 belong to class II sub-genotype XXI.1.2 (7.6% nucleotide distance). The viruses from sub-genotypes XXI.1.1, XXI.2, and XXI were more distant and showed 9.2%, 12%, and 14.7% nucleotide divergence, respectively. The viruses from genotype VI, commonly associated with ND in pigeons, were also very divergent, with 12.4% nucleotide distance. The viruses from genotype XX were more than 10% distant (11% nucleotide distance). As per the latest nomenclature system [11], the phylogenetic analysis classified BD-P01 into class II genotype XXI (Figure 1, Supplementary Figure S1). Among the three sub-genotypes of genotype XXI (i.e., XXI.1.1, XXI.1.2, and XXI.2), the studied Bangladeshi pigeon isolate clustered with sub-genotype XXI.1.2 (former VIm) NDV isolated from pigeons in Pakistan during 2014–2018.

**Figure 2:**
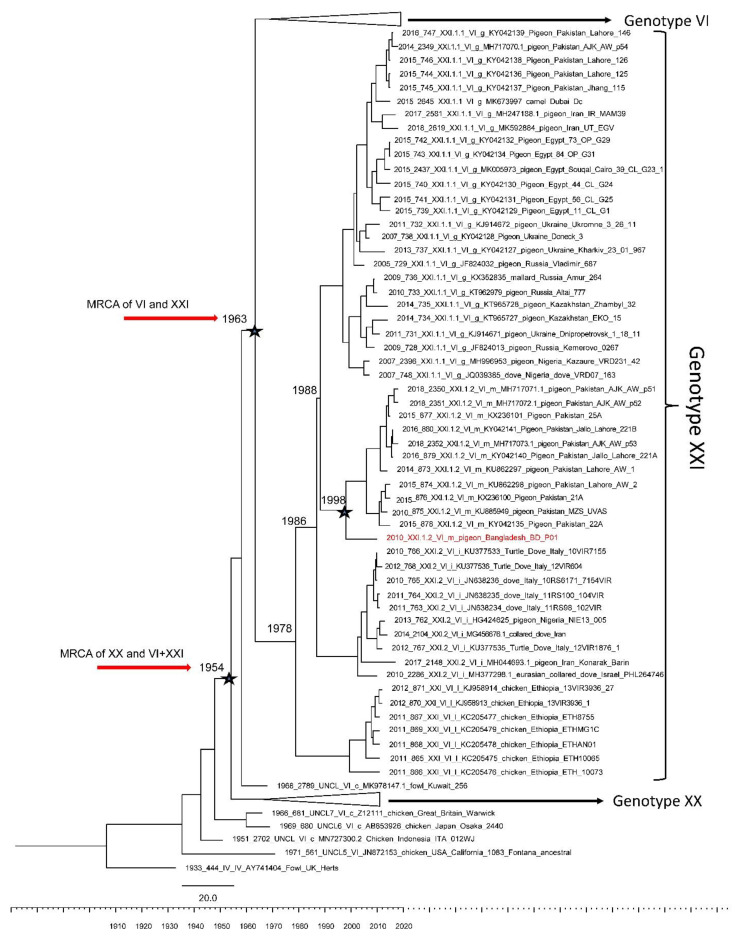
Maximum Clade Credibility (MCC) tree from Bayesian analysis utilizing a sub-set of all full fusion gene sequences (*n* = 383) of genotypes VI, XX, and XXI. The analysis was conducted by the Bayesian Markov Chain Monte Carlo (BMCMC) method implemented in BEAST v1.10.4. The Bangladeshi isolate BD-P01 is highlighted in red font.

In order to estimate the time to most recent common ancestor (tMRCA) between the studied isolate and related viruses, we used a BMCMC approach. The Bayesian tree confirmed the topology of the Maximum Likelihood Analysis. Maximum Clade Credibility trees revealed that BD-P01 and the viruses from sub-genotype XXI.1.2 have evolved from an ancestor that existed during the late 1990s (Figure 2). Genotype XXI and genotype VI emerged from common ancestors during the early 1960s (Figure 2), which is in agreement with previously reported estimates [59,60]. Interestingly, genotypes VI and XXI share common ancestors with viruses that were previously designated as sub-genotype VIc and were recently re-classified as genotype XX. Genotype XX contains predominantly chicken viruses isolated in the 1980s and the 1990s and group together with unclassified chicken viruses from the 1950s and 1960s. The estimated tMRCA suggests that the common ancestors between these viruses and those from genotypes VI and XXI circulated during the late 1950s.

### 3.3. Pathogenicity of BD-P01 for Chickens

#### 3.3.1. Clinical Signs and Mortality

Five-week-old *Sonali* chickens were infected with the BD-P01 isolate. Clinical signs, gross pathology, and microscope lesions were recorded. The morbidity and mortality data of the infected chickens are summarized in Table 2. The post-inoculation survival is depicted by a survival curve in Supplementary Figure S2. The infected chickens did not show any clinical signs during the first 6 dpi. At 7 dpi, four of the infected chickens showed sudden onset of lethargy and paralysis, and three chickens died. At 8 dpi, three more chickens died, and five more chickens showed neurological signs, with paralyses of legs and wings. At 9 dpi, 10 chickens died, and the remaining three chickens were euthanized due to severe clinical signs. The average death time for the group was 7.85 days. The birds in the control group remained healthy throughout the duration of the study period.

#### 3.3.2. Gross Lesions

**Figure 3:**
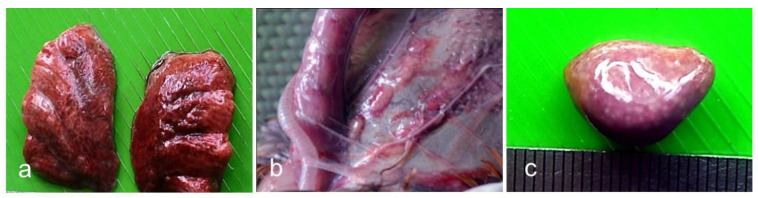
Gross lesions of chickens infected with the pigeon isolate of NDV BD-P01. (**a**) Congestion of lungs at 9 dpi; (**b**) congestion of thymus at 9 dpi; and (**c**) necrosis of the spleen at 9 dpi.

No gross lesions were found at 1, 2, 3, and 5 dpi. At 7 dpi, slight congestion was found in the lungs of one dead chicken. At 9 dpi, there was slight congestion in the thymus and necrosis in the spleen of two dead chickens. On the same day, slight congestion was also recorded in the lungs of three dead chickens. In addition, one dead bird had haemorrhages in the proventriculus. Common gross lesions of chickens infected with the BD-P01 NDV are presented in Figure 3.

#### 3.3.3. Histopathological Changes

Tissue samples from both NDV-infected and control chickens were collected at necropsy and examined after haematoxylin and eosin staining. In the respiratory system, congestion and haemorrhages in the lung of the infected chickens were observed at 7 dpi. In addition, proliferation of pneumocytes II was seen at 9 dpi. No significant lesions were observed in the trachea of the infected chickens until 9 dpi, when there were haemorrhages and desquamation of mucosal epithelium.

The proventriculus showed congestion and slight haemorrhages at 7 dpi. In addition, infiltration of macrophages in the lamina propria was found at 9 dpi. In the intestine, congestion and necrosis of the mucosa with sloughing of the epithelium were seen in two birds at 5 dpi. The liver of two infected chickens showed few necrotic foci at 3 dpi and slight congestion at 9 dpi. The kidney of the infected chickens showed congestion and tubular necrosis with karyorrhexis at 5 dpi and onward. Slight congestion and haemorrhages were noticed in the epicardium at 5 and 7 dpi.

**Figure 4:**
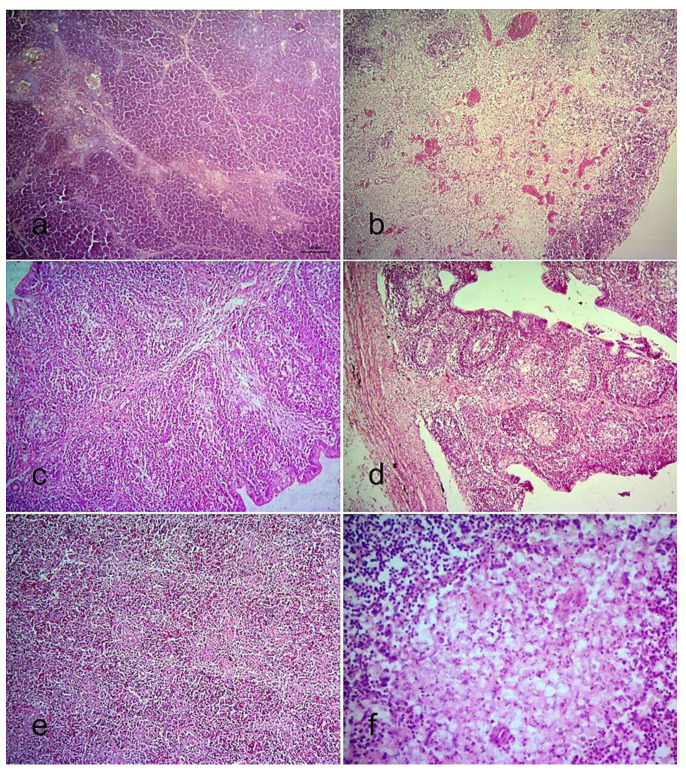
Histopathological changes in different lymphoid tissues of chickens infected with BD-P01. (**a**) Section of the thymus, (**c**) bursa of Fabricius, and (**e**) spleen of uninfected chickens; (**b**) lymphoid depletion and congestion in the thymus at 5 dpi, (**d**) severe lymphoid depletion in the bursa of Fabricius at 7 dpi; (**f**) lymphoid necrosis in the spleen at 7 dpi. H & E stain, 10× (**a**–**e**), 40× (**f**).

Congestion was observed in the Harderian gland at 1 and 2 dpi in all infected chickens. The thymus, bursa of Fabricius, and spleen of the uninfected control chickens showed normal structure (Figure 4a,c,e), whereas the infected chickens showed severe lymphoid depletion (Figure 4b,d,f). In all infected chickens, lymphoid depletion in the thymus started at 5 dpi (Figure 4b) and became severe at 7 to 9 dpi. The bursa of Fabricius of all infected chickens showed mild depletion of lymphocytes in the bursal follicles at 2 dpi, which steadily increased, leading to significant depletion at 5 and 7 dpi (Figure 4d). Follicular atrophy and fibroplasia in the bursa of Fabricius were found at 9 dpi. In the spleen, slight depletion of lymphocytes was observed at 2 and 3 dpi in all infected chickens, followed by multifocal lymphoid necrosis at day 5 and afterwards (Figure 4f).

**Figure 5:**
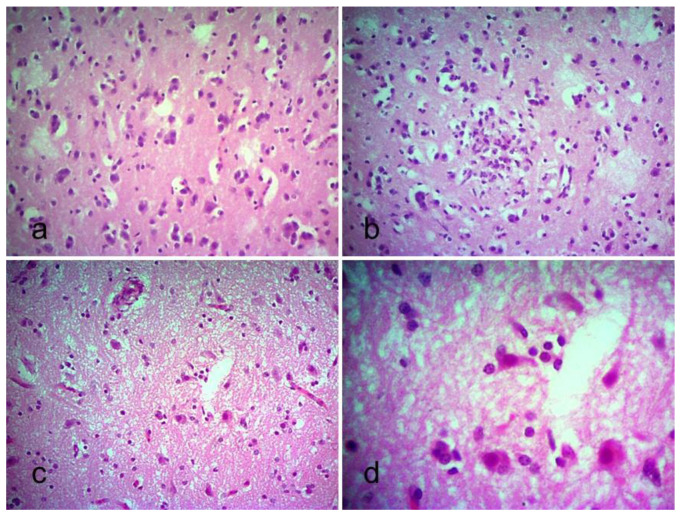
Section of the brain of chickens infected with the pigeon isolate BD-P01. (**a**) Microglial cells and oligodendrocytes proliferation (40×); (**b**) nodular proliferation of oligodendrocyte (40×); (**c**) satellitosis around degenerating neuron (40×); (**d**) higher magnification showing satellitosis around degenerating neuron (100×). H & E stain.

Lesions were also found in the brain of the infected chickens. Mild to moderate proliferation of glial cells (Figure 5a) was seen at 2 dpi, which gradually increased in the course of time, leading to focal nodular aggregation (Figure 5b). There was also satellitosis along with neuronal degeneration (Figure 5c,d) and neuronal necrosis in the infected chickens.

## 4. Discussion

Here, we sequenced the full-length fusion gene coding sequence of a pigeon-derived NDV isolated in Bangladesh in 2010. The isolate was characterized as a member of genotype XXI, and this is the first report of a virus from this genetic group in the country. Experimental infection of chickens revealed that the virus is velogenic for chickens, which is not typical for most NDV isolates from pigeons. The performed complete fusion gene sequencing and molecular epidemiological and pathogenicity characterization provide valuable information on virus distribution, diversity, and evolution, which will support future ND studies.

In this study, we provide the first complete F-gene sequence of a pigeon-derived NDV from Bangladesh (BD-P01). The characterized virus belongs to sub-genotype XXI.1.2 of genotype XXI and is most closely related to NDV isolated from pigeons in Pakistan during 2010–2018. However, the high nucleotide distance between BD-P01 and the viruses from Pakistan (7.6%) suggests that they did not evolve from each other or from a recent common introduction. The BD-P01 virus demonstrates high genetic diversity (>5% nucleotide distance to existing groups of genotype XXI); however, it cannot be designated into a new sub-genotype, as at least four independent isolates are needed per current classification criteria [11]. BD-P01 was identified in 2010, and no similar viruses have been reported since then. For this reason, the isolate may belong to a diverse group of viruses that is underreported due to scarcity of sampling/sequencing efforts. Alternatively, the virus could represent a group that does not naturally circulate anymore.

The studied pigeon-derived BD-P01 was virulent but not fully adapted to chickens. The experimental infection of 5-week-old chickens demonstrated that chickens were susceptible to infection with BD-P01, and the virus induced high mortality in this species. The observed clinical signs were typical for Newcastle disease and, together with the estimated MDT of 40.8 h, confirmed the velogenic nature of the virus. While the challenge virus readily infected chickens through the natural route of exposure, it seems it was not entirely adapted to poultry. Multiple studies show that experimental infection of naïve chickens with chicken-adapted NDV results in early onset of disease (usually by 5 dpi), often as early as 1–2 dpi, with marked gross lesions [4,28,61]. The gross lesions in chickens following infection with BD-P01 were scarce, including mostly congestion and hemorrhages in individual birds. The first clinical signs in the present study were observed at 7 dpi, and the birds’ mean death time was just under 8 days. However, once clinical signs were present, the disease rapidly developed, and all birds died by 10 dpi. The observed clinical signs and microscopic lesions were suggestive that BD-P01 is of neurotropic NDV pathotype. Clinical signs included mostly lethargy and wing and leg paralyses. There were remarkable neuronal lesions comprising gliosis and neuronal degeneration. Similar neuronal lesions in chickens following infection with pigeon-derived NDV isolates have also been reported earlier [27,32]. Pigeon-derived NDV have been documented to cause neurological signs in chickens [62,63]. A limitation of the current study is that commercial birds were used in the animal experiment. If these birds had underlying asymptomatic infections (e.g., mycoplasma, infectious bursal disease, or Marek’s disease), the latter could have exacerbated the clinical presentation after inoculation with BD-P01. However, these birds were raised in isolation and were apparently healthy. No gross or microscopic lesions were observed in the control group, indicating that the observed clinical signs and pathologic lesions in the experimental group resulted from the BD-P01 challenge. The performed study provides evidence of the potential of the BD-P01 virus to cause clinical disease in commercial chickens.

The studied BD-P01 isolate may be a chicken-origin virus in process of adaptation to pigeons. It has been previously demonstrated that over time pigeon NDV isolates appear to increase their adaptation and virulence in pigeons with corresponding decrease in adaptation and virulence for chickens [4,23]. This could explain the severity of clinical signs that BD-P01 caused in pigeons and the delay of disease onset and scarcity of gross lesions in chickens. Experimental pathogenicity studies reveal that most NDV isolated from pigeons are highly virulent for pigeons and of moderate or low virulence for chickens [22,23,24,25,27,28], which is probably a result of longer adaptation to pigeons for these viruses as compared to BD-P01 (as it emerged in the last 20 years). Of note, serial passages of pigeon-origin NDV in chickens have shown to increase their pathogenicity for chickens [32,34,64]. Indeed, recent reports have suggested that the viruses from genotype XXI have evolved from chicken-adapted viruses, further supporting our findings. Naguib et al. suggested that the viruses of genotypes VI and XXI are descendants of viruses that circulated in poultry in the 1950s and 1960s [65]. Similarly, Afonso discussed that the pigeon NDV lineages (i.e., genotypes VI and XXI) may have originated from a chicken-adapted lineage (i.e., older genotype XX, consisting of chicken viruses) [66].

The estimated nucleotide distances and performed phylogenetic and Bayesian analyses provide sufficient evidence to suggest a continuous evolution of genotype XXI NDV. Genotype XXI is relatively young and has been identified only recently. The first reported viruses were isolated in 2005. Despite their recent identification, the sub-genotypes of genotype XXI show significant diversity, with nucleotide distances between them ranging from 7.6% to 14.8% (Table 2). Such high genetic distance cannot be explained with local evolution over the last 16 years during which all available viruses from genotype XXI have been reported. The estimated tMRCA suggests that genotypes XXI and VI evolved from a common ancestor that circulated in the early 1960s. A period of almost 60 years is in agreement with the genetic diversification that is observed between the groups within genotype XXI and also between genotypes VI and XXI. The estimates of tMRCA presented here align with previous studies utilizing large sequence datasets [59,60,67].

Where and how the viruses from genotype XXI have been maintained over the years until first identified in 2005 is puzzling. The hypotheses of maintenance in immune poultry or in wild birds are viable. It has previously been suggested that NDV can be maintained in vaccinated poultry [68,69]. While vaccinated birds do not show clinical signs, they still shed the virus after infection with NDV [70,71]. The birds from the family *Columbidae* have been documented as natural reservoirs of some NDV [4], and it is not unlikely that the viruses from genotype XXI have been harbored in these species. These viruses may have remained undetected and unreported due to scarcity (or lack thereof) of surveillance efforts. Columbid birds are commonly not included in routine monitoring programs, and NDV from these birds are mostly identified during massive die-offs or research studies using convenience samples.

## 5. Conclusions

Overall, our study provides valuable information on NDV of genotype XXI that will facilitate future investigation of NDV epidemiology. The results reported here highlight the need to include synanthropic and wild bird populations in NDV-monitoring efforts and not focus only on poultry. The identification of a pigeon-derived virus that is virulent to chickens emphasizes the importance of biological characterization of such NDV isolates and that the assumption that they are generally of low or moderate virulence to chickens should not be over-trusted. Additional studies to identify and characterize more genotype XXI NDV will aid to further elucidate the evolution and these viruses. A vaccination-challenge study with currently utilized vaccines is warranted to reveal if they provide protection against introduction of BD-P01 into poultry.

## Figures and Tables

**Table 1:** Estimates of evolutionary distance between the studied BD-P01 virus and different sub/genotypes of class II Newcastle disease viruses that have been associated with pigeons.

Sub/Genotype	No. of Base Substitutions per Site ^a^					
	BD-P01	XXI.1.1	XXI.1.2	XXI.2	XXI	XX
BD-P01						
XXI.1.1	0.092					
XXI.1.2	0.076	0.093				
XXI.2	0.120	0.105	0.124			
XXI	0.147	0.125	0.139	0.148		
XX	0.110	0.096	0.111	0.118	0.127	
VI	0.124	0.108	0.125	0.131	0.148	0.102

^a^ The number of base substitutions per site from averaging over all sequence pairs between groups are shown. Analyses were conducted using the Maximum Composite Likelihood model [49]. The rate variation among sites was modelled with a gamma distribution (shape parameter = 1). The analysis involved 383 nucleotide sequences. All positions containing gaps, and missing data were eliminated. There were a total of 1652 positions in the final dataset. Evolutionary analyses were conducted in MEGA6 [48].

**Table 2:** Morbidity and mortality in chickens following infection with BD-P01.

Days Post Infection	No. of Birds under Observation	No. of Birds Sick	No. of Birds Died	No. of Birds Sacrificed (Sick/Normal)
1	31	0	0	3
2	28	0	0	3
3	25	0	0	3
5	22	0	0	3
7	19	4	3	-
8	16	5	3	-
9	13	3	10	3

## Data Availability

All authors agree that the data presented in this study are openly available through MDPI publisher platform or others without any restriction.

## Supplementary Materials

The following are available online at https://www.mdpi.com/article/10.3390/v13081520/s1, Figure S1: Phylogenetic analysis based on full-length nucleotide sequence of the fusion gene of isolates representing Newcastle disease virus class II (*n* = 1952). Figure S2: Survival of 35-day-old *Sonali* chickens after inoculation with BD-P01.
