# Supplementary figures and images for: A Pigeon-Derived Sub-Genotype XXI.1.2 Newcastle Disease Virus from Bangladesh Induces High Mortality in Chickens
Source: Viruses. 2021 Aug 1;13(8):1520. doi: 10.3390/v13081520 (PMC8402815; doi:10.3390/v13081520)

Supplemental Figure S2. Survival of 35-day-old Sonali chickens after inoculation with BD-P01.

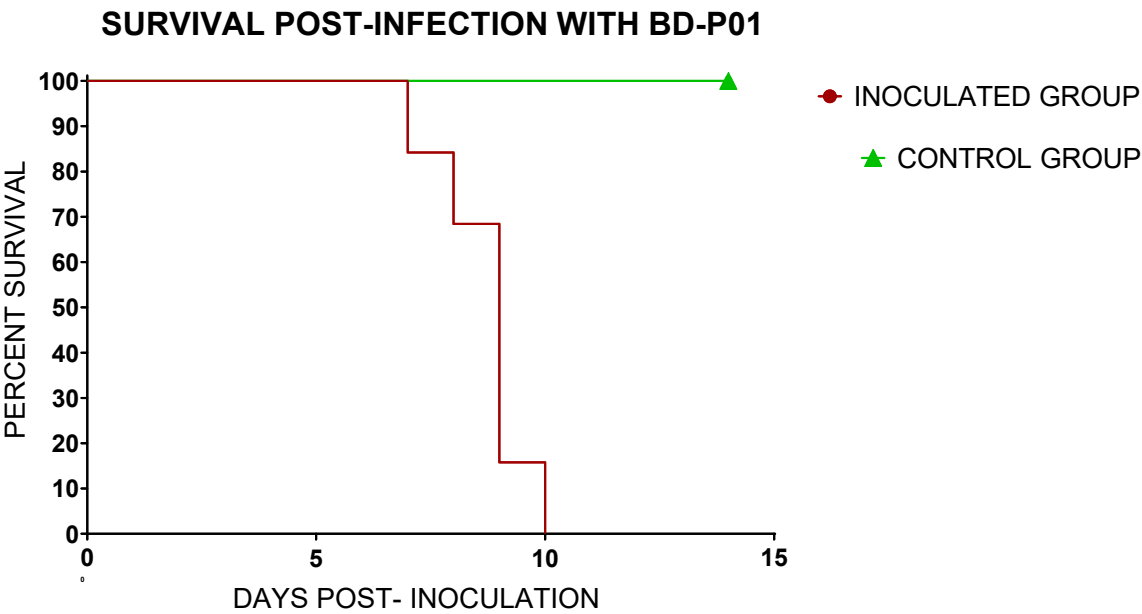

Supplement: Supplementary file 1 [file viruses-13-01520-s001.zip › Suppl. Fig. 2.pdf]
